# Gastrodin prevents homocysteine‐induced human umbilical vein endothelial cells injury via PI3K/Akt/eNOS and Nrf2/ARE pathway

**DOI:** 10.1111/jcmm.16073

**Published:** 2020-12-15

**Authors:** Jiyu Chen, Yanli Huang, Xiaochuan Hu, Xiaohong Bian, Sihui Nian

**Affiliations:** ^1^ School of Life Science and Technology China Pharmaceutical University Nanjing China; ^2^ Department of Occupational disease Qingdao Central Hospital Shandong China; ^3^ Institute of Modern Chinese Medicine School of Pharmacy Wannan Medical College Wuhu China

**Keywords:** Cav‐1/eNOS, Gastrodin, homocysteine, Nrf2, oxidative stress

## Abstract

In this study, we investigated the protective effects of gastrodin (Gas) against homocysteine‐induced human umbilical vein endothelial cell (HUVEC) injury and the role of the phosphoinositide 3‐kinase (PI3K)/threonine kinase 1 (Akt)/endothelial nitric oxide synthase (eNOS) and NF‐E2‐related factor 2 (Nrf2)/antioxidant response element (ARE) pathways. We stimulated cells with homocysteine (1 mmol/L, 24 hours) and tested the effects of gastrodin (200‐800 μg/mL) on cell viability and the production of malondialdehyde (MDA), lactate dehydrogenase (LDH) and reactive oxygen species (ROS). Then, Nrf2 distribution in the cytoplasm and nucleus as well as the expression of enzymes downstream of Nrf2 was determined. Furthermore, we analysed the expression of bax, bcl‐2 and cleaved caspase3, and assessed the involvement of the PI3K/Akt/eNOS pathway by Western blots. Finally, we tested the vasoactive effect of gastrodin in thoracic aortic rings. The results showed that gastrodin decreased MDA, LDH and ROS production and increased cell viability, NO production and relaxation of thoracic aortic rings. Moreover, the protective effects of Gas on NO production and relaxation of thoracic aortic rings were blocked by L‐NAME but enhanced by Cav‐1 knockdown, and MK‐2206 treatment abolished the effect of Gas on the ROS. In addition, treatment with gastrodin increased Nrf2 nuclear translocation, thus enhancing the expression of downstream enzymes. Finally, gastrodin increased the expression of PI3K, p‐Akt, and eNOS and decreased Cav‐1 protein expression. In conclusion, our study suggested that gastrodin may protect HUVECs from homocysteine‐induced injury, and the PI3K/Akt/eNOS and Nrf2/ARE pathways may be responsible for the efficacy of gastrodin.

## 1 INTRODUCTION

Recently, experimental and clinical studies have proven that homocysteine (Hcy) plays an important role in the development of various cardiovascular diseases.^1^ Hcy can cause vascular endothelial cell damage and enhance oxidative stress,^2^ which could reduce the production and bioavailability of endothelial‐derived vasodilators such as nitric oxide (NO) as well as promote extracellular matrix accumulation and smooth muscle cell proliferation. These changes can lead to vascular endothelial dysfunction, decreased endothelium‐dependent vasorelaxation and high blood pressure.^3^ Cav‐1, the principal coat protein of caveolae, is a key regulator of NO generated by eNOS.^4^ Cav‐1 regulates the eNOS/NO pathway through the caveolin scaffold domain (CSD).^5^ Interaction between eNOS and CSD leads to inhibition of eNOS activity, resulting in decreased NO production.^6^ Hcy decreases NO production through the regulation of Cav‐1 expression and the interaction of Cav‐1 and eNOS.^7^ In addition, Hcy can induce reactive oxygen species (ROS) production, subsequently leading to apoptosis.^8^ Moreover, the Hcy‐induced reduction of the Nrf2‐dependent antioxidant defence system suppressed antioxidant enzymes downstream of Nrf2.^9^, ^10^ Hence, research has indicated that decreasing the accumulation of ROS and restoring NO production could inhibit Hcy‐induced injury.^11^

Gastrodin (Gas, PubChem CID: 115 067) is a major active component in *Gastrodia elata* Blume, which belongs to a species in the genus *Gastrodia* (family *Orchidaceae*) and is commonly known as Tianma in Chinese. In China, traditional Chinese physicians have used *G. elata* Blume for the treatment of cerebrovascular and cardiovascular diseases, such as stroke, migraine, cardiac hypertrophy, fibrosis and hypertension.^12^, ^13^ Gastrodin injection significantly decreased systolic pressure and pulse pressure in elderly patients with refractory hypertension after one course of treatment.^14^ This molecule is used extensively in Chinese medicine and has analgesic and sedative effects. Gas has also been confirmed to have anti‐inflammatory and antioxidant activity by suppressing proinflammatory genes and removing oxygen free radicals.^15^ In addition, Gas exerted a protective effect on Schwann cells through the regulation of the PI3K/Akt signalling pathway, which plays an important role in the activation of eNOS and Nrf2.^16^

However, the effect of Gas on Hcy‐induced endothelial dysfunction remains unclear. Based on the pharmacological activities of Gas in many cardiovascular diseases, we have been suggested that Gas would exert a protective effect on Hcy‐induced endothelial dysfunction through the PI3K/Akt pathway. Therefore, in this study, we explored whether Gas protects against Hcy‐induced vascular endothelial cell injury through the regulation of the PI3K/Ake/eNOS and Nrf2/ARE pathways. This study may provide a basis for application of Gas in the treatment of Hcy‐induced vascular endothelial injury.

## 2 MATERIALS AND METHODS

### 2.1 Cell culture

Human umbilical vein endothelial cells (HUVECs) were purchased from ScienCell and cultured in complete ECM medium (ScienCell, CA, USA) that contained 5% FBS, 1% endothelial cell growth supplement, 100 U/mL penicillin, and 0.1 mg/mL streptomycin (Beyotime Biotechnology, Shanghai, China) at 37°C under 5% CO_2_. The experimental groups were designed as follows: (a) Control group: normal cells without Hcy (1 mmol/L) and Gas (200‐800 μg/mL) treatment for 24 hours; (b) Model group: cells treated with Hcy (1 mmol/L) for 24 hours; (c) Protective group: cells treated with Hcy (1 mmol/L) and Gas (200‐800 μg/mL) for 24 hours. Gastrodin (IUPAC: (2R,3S,4S,5R,6S)‐2‐(hydroxymethyl)‐6‐[4‐(hydroxymethyl)phenoxy]oxane‐3,4,5‐triol) was purchased from Chengdu Must Bio‐Technology *Co.,* Ltd. (≥98%, A0138, Chengdu, China) and dissolved in PBS. Hcy was purchased from Sigma (≥98%, H4628, Missouri, USA) and dissolved in PBS. For the Akt inhibition experiment, the Akt inhibitor MK‐2206 was purchased from Selleckchem (S1078, Houston, USA) and added to cell culture medium with Gas and Hcy for 24 hours.

### 2.2 Assessment of cell viability

HUVECs were plated in 96‐well plates at a density of 1 × 10^4^ cells/well. After reaching 80% confluence, the cells were treated with Hcy (1 mmol/L) in the absence or presence of Gas (50‐800 µg/mL) for 24 hours. An MTT assay was performed to assess cell viability. Briefly, 20 μL of 5 mg/mL MTT (40201ES72; Yeasen, Shanghai, China) was added to each well in culture medium and incubated at 37°C for 4 hours. Next, the culture medium was removed, the remaining crystals were dissolved in 200 μL of DMSO, and plates were incubated at room temperature for 10 minutes on a shaking table. The absorbance at 492 nm was measured using a microplate reader (Model 680; Bio‐Rad, CA, USA). The cellular ATP content was also used for the detection of cell viability. The CellTiter‐Lumi^TM^ luminescent Cell Viability Assay Kit (Beyotime, Shanghai, China) was used for quantification of ATP. Briefly, 100 μL of reaction solution was added to 96‐well plates and incubated at room temperature for 10 minutes. The luminescent signal was recorded on a Thermo Varioskan Flash microplate reader.

### 2.3 Biochemical analysis

HUVECs were plated in 24‐well plates at a density of 1.3 × 10^5^ cells/well. The cultured cells were treated with Gas (50‐800 μg/mL) and Hcy (1 mmol/L) for 24 hours after plating. For eNOS inhibition, 24 hours after plating, HUVECs were pre‐treated with L‐NAME (100 μmol/L) (N5751; Sigma, Missouri, USA) for 30 minutes and then incubated with Hcy (1 mmol/L) in the presence or absence of Gas (50‐800 μg/mL) for 24 hours in complete medium. After 24 hours, HUVEC supernatants were collected, and the MDA and LDH activities were determined using an MDA assay kit (A003‐1‐2; Nanjing Jiancheng Bioengineering Institute, Nanjing, China), LDH assay kit (A020‐2‐2; Nanjing Jiancheng Bioengineering Institute, Nanjing, China) and total antioxidant capacity (T‐AOC) assay kit (S0116; Beyotime, Shanghai, China) according to the manufacturer's instructions.

### 2.4 Evaluation of vascular function

The rats were anaesthetized, and the thoracic aortas were isolated after removal of connective tissue and fat. Then, the thoracic aortas were cut into rings approximately 4 mm wide, and two stainless‐steel triangles were inserted into the lumen of each ring. Then, each ring was set up onto a tension transducer connected with a Powerlab System in gassed (95% O_2_, 5% CO_2_) Krebs solution (mmol/L: NaCl 114, KCl 4.7, CaCl_2_ 2.5, MgCl_2_ 1.2, NaHCO_3_ 25, KH_2_PO_4_ 1.2, glucose 10, pH 7.4) at 37℃ under a resting tension of 1.0 g for 2 hours to equilibrate. After equilibration, the aortic rings were constricted with 80 mmol/L KCl to test its availability. Then, the rings were washed with Krebs solution to a basic tension of 1.0 g. The viability of the endothelium was evaluated by the addition of acetylcholine (ACh, 1 × 10^−5^ mol/L, Sigma, A6625) to induce >70% relaxation. The rings were pre‐treated by norepinephrine (1 × 10^−6^ mol/L) to reach the plateau phase, and Gas or ACh was added to the bath in batches at 5 minutes.

The experimental groups were treated as follows: (a) Control group: rings were treated with Gas or ACh; (b) Model group: rings were pre‐treated with Hcy (1 mmol/L) for 30 minutes; (c) Inhibitor group: rings were pre‐treated with L‐NAME (100 μmol/L) and then treated with Hcy, followed by Gas or ACh.

### 2.5 Detection of ROS and NO

HUVECs were cultured in 6‐well plates (2 × 10^5^ cells/well) and treated with Hcy (1 mmol/L) in the presence or absence of Gas (200, 400, 800 μg/mL) for 24 hours. Then, the cells were incubated with dihydroethidium hydrochloride (DHE, 2 μmol/L) for ROS detection or 4‐amino‐5‐methylamino‐2',7'‐difluorofluorescein (DAF‐FM, 5 μmol/L) for NO detection in the cell incubator at 37℃ for 30 minutes. The cells were washed twice with PBS and detected by inverted fluorescence microscopy (Carl Zeiss, Axio Vert. A1, Oberkochen, Germany) at 535 nm excitation and 610 nm emission or 495 nm excitation and 515 nm emission. The mean fluorescence was quantified using ImageJ software (NIH, USA).

The NO contents were measured using an NO assay kit (S0021S; Beyotime Biotechnology, Shanghai, China). Briefly, 50 μL of culture medium from HUVECs under different treatments and 50 μL of Griess reagents I and II were added to a 96‐well plate at room temperature for 30 minutes, and the NO level was determined by a microplate reader (Model 680; Bio‐Rad, CA, USA) at 540 nm.

### 2.6 Immunofluorescence staining

HUVECs were cultured in confocal dishes (2 × 10^5^ cells/well) and treated with Hcy (1 mmol/L) in the presence or absence of Gas (200, 400, 800 μg/mL) for 24 hours. After the treatment, the cells were washed twice with PBS and then fixed with 4% (w/v) paraformaldehyde for 15 minutes at room temperature. Then, the cells were washed with PBS twice, and subsequently, the cells were permeabilized with 0.3% Triton X‐100 for 10 minutes. Then, 0.03% Triton X‐100 and 5% BSA in PBS were added to the dishes to block for 30 minutes at room temperature. Next, the cells were incubated with Nrf2 antibody (1:100, Santa Cruz, sc‐365949, CA, USA) diluted in PBS containing 0.3% BSA at 4°C overnight. Then, the cells were washed twice with PBS and incubated with secondary antibody (1:500, Abcam, ab150116, Cambridge, UK) diluted in PBS for 2 hours at 37°C. Finally, the images were captured by a laser confocal fluorescence microscope (FV1000, Olympus, Japan) at a thickness of 1 μm.

### 2.7 Quantitative real‐time RT‐PCR

HUVECs were cultured in 6‐well plates (2 × 10^5^ cells/well) and treated with Hcy (1 mmol/L) in the presence or absence of Gas (200, 400, 800 µg/mL). Total RNA was isolated using TransZol Up reagent (1 mL/well, ET111‐01; TransGen Biotech, Beijing, China). One microgram of total RNA was reverse transcribed with First‐strand cDNA Synthesis SuperMix (AT341‐01; TransGen Biotech, Beijing, China). Real‐time PCR was performed using TransStart Green qPCR SuperMix UDG (AQ111‐01; TransGen Biotech, Beijing, China) based on the manufacturer's instructions.

The primer sequences for HO‐1 were 5'‐CAGGATTTGTCAGAGGCCCTGAAGG‐3' and 5'‐TGTGGTACAGGGAGGCCATCACC‐3'; those for SOD‐1 were 5'‐GGTGGGCCAAAGGATGAAGAG‐3' and 5'‐CCACAAGCCAAACGACTTCC‐3'; and those for catalase were 5'‐TGGAGCTGGTAACCCAGTAGG‐3' and 5'‐CCTTTGCCTTGGAGTATTTGGTA‐3'. The mRNA of β‐actin (forward: CATGTACGTTGCTATCCAGGC, reverse: CTCCTTAATGTCACGCACGAT) was quantified as an endogenous control.

### 2.8 Subcellular fractionation

Nuclear and cytosolic proteins were isolated from HUVECs treated with Hcy (1 mmol/L) in the absence or presence of Gas (200, 400, 800 µg/mL) using a nuclear extract kit (P0027; Beyotime) according to the manufacturer's instructions. Briefly, the cells were collected using a cell scraper and centrifuged at 400 g for 5 minutes at 4°C. After centrifugation, the supernatant was discarded. Then, 200 μL of cytoplasmic protein extract solution with 1 mmol/L PMSF was added and vortexed for 5 seconds. After that, the samples were incubated at 4℃ for 10 minutes. The samples were then vortex‐mixed and centrifuged for 5 minutes at 12 000 g at 4℃. The supernatant as the cytoplasmic protein fraction was collected. Next, the precipitate was dissolved in the nuclear extract solution, and then, the samples were vortex‐mixed and incubated in an ice bath for 10 minutes and shaken every 2 minutes. Finally, the samples were centrifuged at 4°C for 10 minutes at 12 000 g, and the supernatant was used for nuclear protein extraction.

### 2.9 Western blot analysis

HUVECs treated with Hcy (1 mmol/L) in the absence or presence of Gas (200, 400, 800 µg/mL) were washed 3 times with ice‐cold PBS and lysed with RIPA lysis buffer (P0013B; Beyotime Biotechnology) supplemented with 1 mmol/L PMSF (ST506; Beyotime Biotechnology). The lysate was centrifuged at 13 800 g and 4°C for 15 minutes. Protein concentrations were measured by a BCA kit (P0010S; Beyotime Biotechnology). Equal amounts of protein were separated by 10% SDS‐PAGE and transferred into PVDF membranes. Having been blocked in 5% fat‐free milk for 2 hours, the membranes were probed with primary antibodies as follows: anti‐eNOS (Santa Cruz, sc‐376751), anti‐phospho‐eNOS (Ser 1177) (Santa Cruz, sc‐81510), anti‐Cav‐1 (Santa Cruz, sc‐53564), anti‐bax (Cell Signaling Technology, 5023S), anti‐bcl‐2 (Cell Signaling Technology, 15071S), anti‐cleaved caspase 3 (Cell Signaling Technology, 9664S), anti‐Nrf2 (Santa Cruz, sc‐365949), anti‐HO‐1 (Santa Cruz, sc‐136960), anti‐SOD‐1 (Santa Cruz, sc‐271014), anti‐catalase (Santa Cruz, sc‐271803), anti‐PI3K p85 (Santa Cruz, sc‐1637), anti‐Akt (Santa Cruz, sc‐81434), anti‐p‐Akt (Santa Cruz, sc‐52940) and anti‐β‐actin (Bioworld, AP0714, Nanjing, China) overnight at 4°C, followed by incubation with goat anti‐rabbit (Bioworld, BS13278) or goat anti‐mouse (Bioworld, BS12478) IgG‐HRP (1:10 000). Bands were detected by ECL (Millipore, WBULS0500, MA, USA). Band intensities were assessed by Quantity One (Bio‐Rad) and normalized to the total protein level or a reference protein.

### 2.10 siRNA transfection

siRNA transfection was performed with Lipofectamine 3000 (Invitrogen, L3000, CA, USA) using Cav‐1 siRNA (5'‐AACCAGAAGGGACACACAG‐3') or control siRNA. siRNA oligos were obtained from GenePharma (Shanghai, China). NO detection assays were performed after 48 hours of siRNA treatment.

### 2.11 Statistical analysis

Data are presented as the mean ± SEM of at least three biological replicates in each experiment. Statistical analysis was performed with GraphPad Prism 6.0, and *P* < 0.05 was considered statistically significant.

## 3 RESULTS

### 3.1 Gas improved cell viability in Hcy‐cultured HUVECs

**FIGURE 1:**
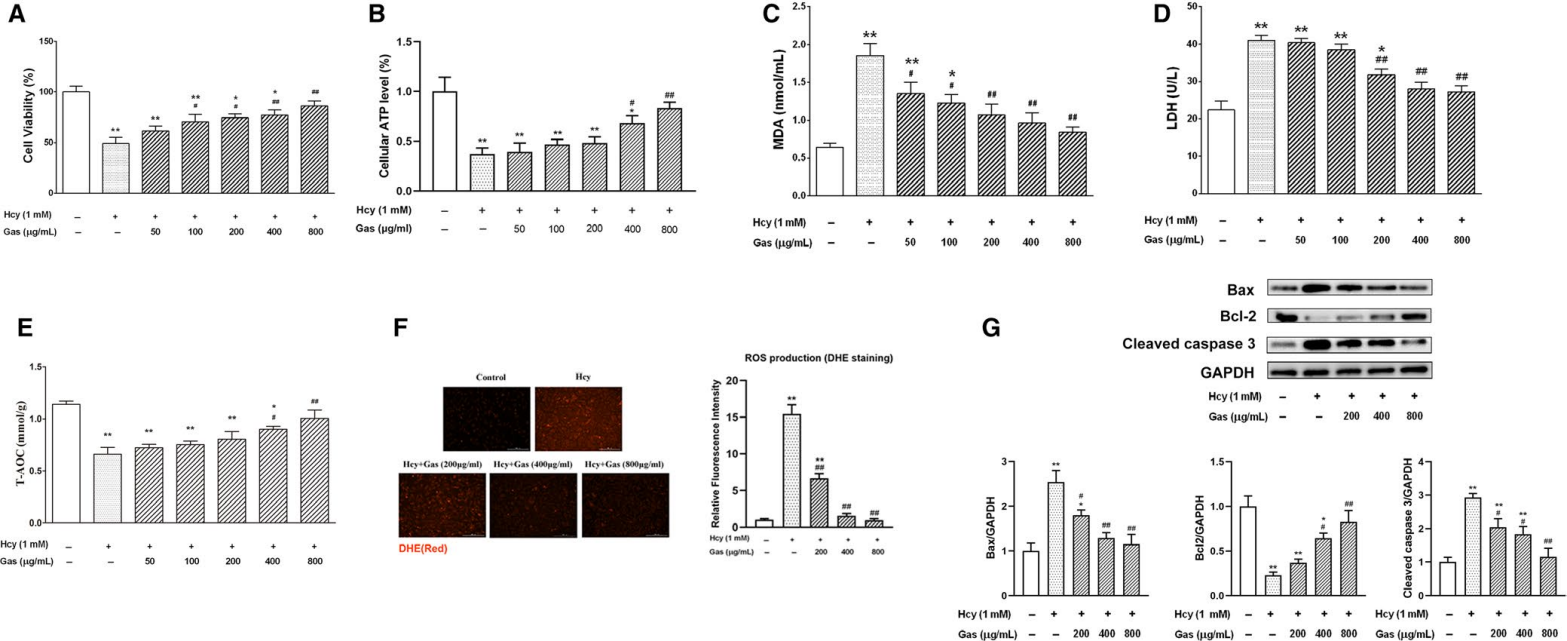
Gas inhibited Hcy‐induced HUVECs oxidative stress and cytotoxicity. HUVECs were incubated with Hcy (1 mmol/L) for 24 h in the absence or presence of Gas (200‐800 μg/mL). (A) Cell viability was measured by MTT assay; (B) ATP content in cells was examined using CellTiter‐LumiTM luminescent Cell Viability Assay Kit. (C‐D) Cells oxidative damage were measured through MDA and LDH detection. (E) T‐AOC were measured by kit assays; (F) Cellular ROS was measured by DHE (2 μmol/L) staining. Imaged at 10 × magnification. Scale bars = 200 μm. Mean fluorescence was quantified using Image J software. (G) Western Blot showed that Gas regulated Hcy‐induced bax, bcl‐2 and cleaved caspase 3 expression. Data are Mean ± SEM (three independent experiments). *P < 0.05 vs. control group. #*P* < 0.05 vs. model group

To assess the effect of Gas on Hcy‐induced injury, we tested the effect of Gas on cell viability. Incubation of HUVECs with Hcy for 24 hours caused a significant decrease in cell viability compared with that of the control group, and this decrease was inhibited by treatment with Gas (Figure 1A). Gas (50‐800 µg/mL) enhanced cell viability with a maximal effect in the 800 µg/mL Gas group, which showed a significant difference compared to the Hcy model group. Cell viability was also determined by measuring the cellular ATP levels in the Hcy‐treated HUVECs with or without Gas. The results showed that Hcy decreased the intracellular levels of ATP in HUVECs, which were restored by Gas in a concentration‐dependent manner, and the 400 and 800 μg/mL Gas groups showed significant differences compared with the model group (Figure 1B). Gas showed no cytotoxicity at concentrations of 50‐800 µg/mL (Supplemental Data, Figure S1).

### 3.2 Gas inhibited the Hcy‐induced increases in ROS levels, MDA content and LDH activity

As MDA and LDH are the most commonly used markers of oxidative damage to lipids and cytotoxicity, respectively, we investigated whether Gas affects these biochemical indicators.^17^, ^18^ The MDA and LDH levels were significantly higher in the model group than in the control group. The MDA levels in the presence of Gas (50‐800 µg/mL) also showed a dose‐dependent reduction with a maximal decrease at 800 µg/mL, and the LDH levels showed significant differences in the 200 to 800 μg/mL Gas groups (Figure 1C, D). In addition, we tested oxidative stress by the detection of T‐AOC, which reflects the capacity of all antioxidants to scavenge free radicals in cells; we found that the stimulation of HUVECs with Hcy decreased T‐AOC and that Gas dose‐dependently increased T‐AOC and induced a significant difference starting at 400 μg/mL (Figure 1E).

ROS play a key role in vascular endothelial dysfunction;^19^ hence, we assessed whether Gas exerts protective effects by decreasing the production of ROS. As shown in Figure 1F, the control group showed the lowest number of red fluorescence spots, and the model group showed the most. Additionally, the number of red fluorescence spots in the presence of Gas showed a dose‐dependent reduction with a maximal decrease at 400 and 800 μg/mL.

### 3.3 Gas inhibited Hcy‐induced HUVEC apoptosis

Hcy‐induced mitochondrial dysfunction and excessive accumulation of ROS in cells lead to apoptosis.^20^, ^21^ To determine the ability of Gas to attenuate the damage in Hcy‐treated HUVECs, we measured the expression of bax, bcl‐2 and cleaved caspase 3, mitochondria‐dependent apoptosis pathway‐related proteins, to explore the antiapoptotic mechanism of Gas in Hcy‐induced HUVECs. The results showed that Hcy increased the expression of bax and cleaved caspase 3, whereas it reduced the expression of bcl‐2. However, Gas treatment effectively increased the expression of bcl‐2 and decreased the expression of bax and cleaved caspase 3, and these parameters showed a significant difference in the 400 and 800 μg/mL groups compared with the Hcy group (Figure 1G).

### 3.4 Gas enhanced the nuclear translocation of Nrf2 and the activation of the Nrf2/ARE pathway

**FIGURE 2:**
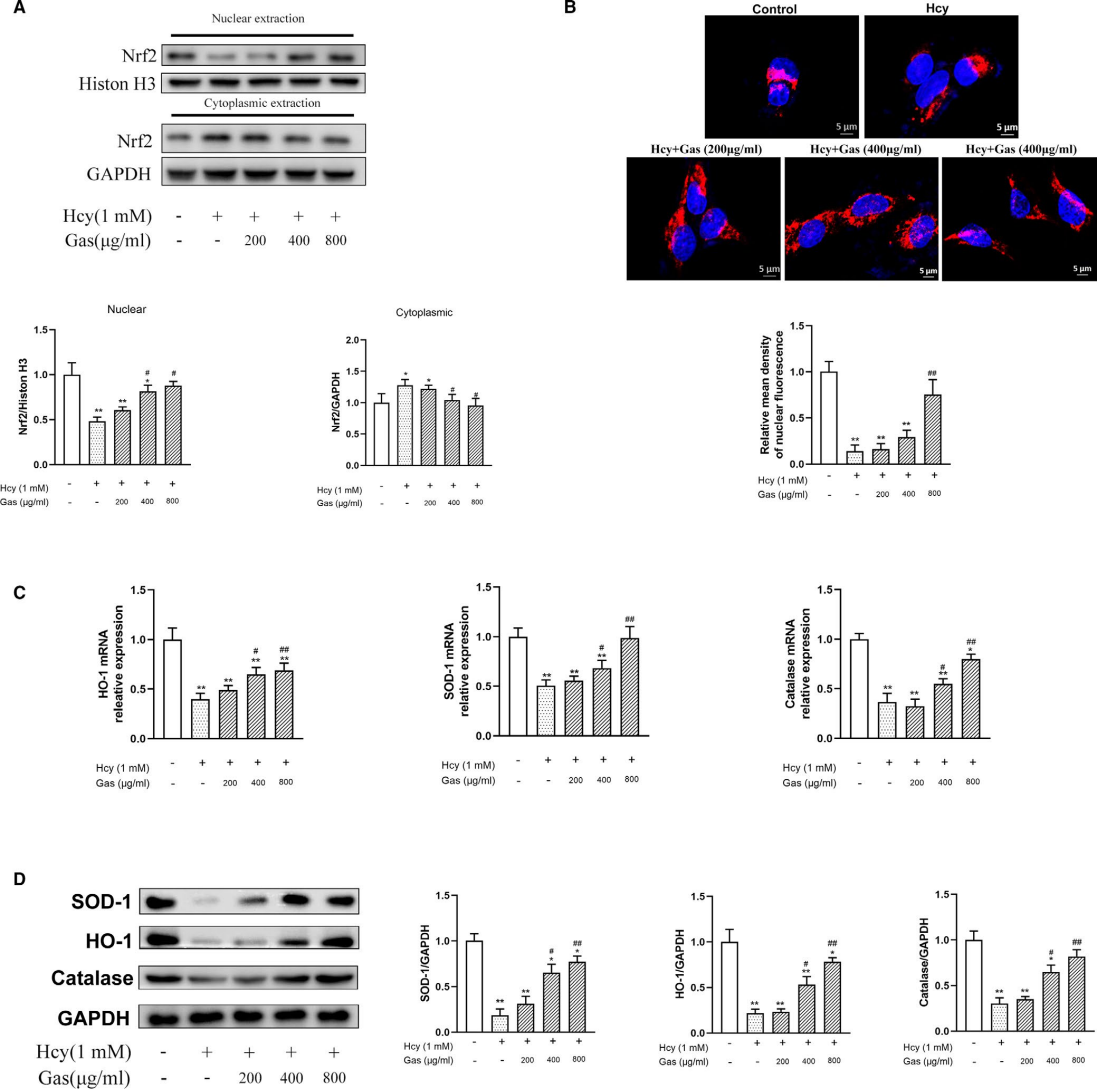
Gas regulated Nrf‐2/ARE pathway against Hcy‐induced endothelial injury. HUVECs were incubated with Hcy (1 mmol/L) for 24 hours in the absence or presence of Gas (200‐800 μg/mL) (A) Gas inhibited Hcy‐induced Nrf2 transferred to cytoplasm; (B) Immunfluorescent staining of HUVECs with anti‐Nrf‐2 antibody (Red) and DAPI (blue). Scale bars = 5 μm. Histograms (mean ± SEM) show Nrf2 fluorescence intensity signal quantification in the nucleus; Gas regulated Nrf‐2/ARE pathway downstream enzymes SOD‐1, HO‐1 and Catalase in mRNA (C) and protein (D) level. Data are Mean ± SEM (three independent experiments). *P < 0.05 vs. control group. #*P* < 0.05 vs. model group

To explore the antioxidant mechanisms of Gas, we studied the Nrf2/ARE pathway. First, we examined the subcellular localization of Nrf2 by isolating nuclear and cytosolic proteins. The results showed that the Hcy‐treated cells had lower Nrf2 levels in the nuclear proteins than the control cells; conversely, Gas dose‐dependently increased the Nrf2 level in the nucleus, and the 400 and 800 μg/mL groups showed a significant difference compared with the Hcy group. Moreover, there was a slight increase in the Nrf2 levels in the cytoplasm in the Hcy‐treated group, and Gas inhibited the increase in Nrf2 in the cytoplasm; in the 800 μg/mL group, this increase was significant compared with that of the Hcy group (Figure 2A, B).

Nrf2 is a transcription factor that can bind to antioxidant response elements in the promoters of genes that code for antioxidative enzymes.^22^ The accumulation of Nrf2 in the nucleus could elevate the expression of enzymes downstream of Nrf2. The Nrf2/ARE pathway could maintain oxidative balance, improve antioxidant performance and alleviate oxidative stress.^23^ For instance, HO‐1, SOD‐1 and catalase are downstream of the Nrf2/ARE pathway and play an important role in oxidative stress.^24^ Hence, we assessed the expression of HO‐1, SOD‐1 and catalase in the Hcy‐treated HUVECs with or without Gas by Western blots and qPCR. The results showed that the stimulation of HUVECs with Hcy decreased the expression of HO‐1, SOD‐1 and catalase; moreover, Gas dose‐dependently increased the expression of HO‐1, SOD‐1 and catalase at the protein and mRNA levels, and all these parameters showed significant differences in the 400 and 800 μg/mL Hcy groups but not in the 200 μg/mL Hcy group, indicating that low‐dose Gas had little effect on the expression of Nrf2/ARE downstream enzymes (Figure 2C, D).

### 3.5 Gas induced an increase in NO in Hcy‐damaged HUVECs

**FIGURE 3:**
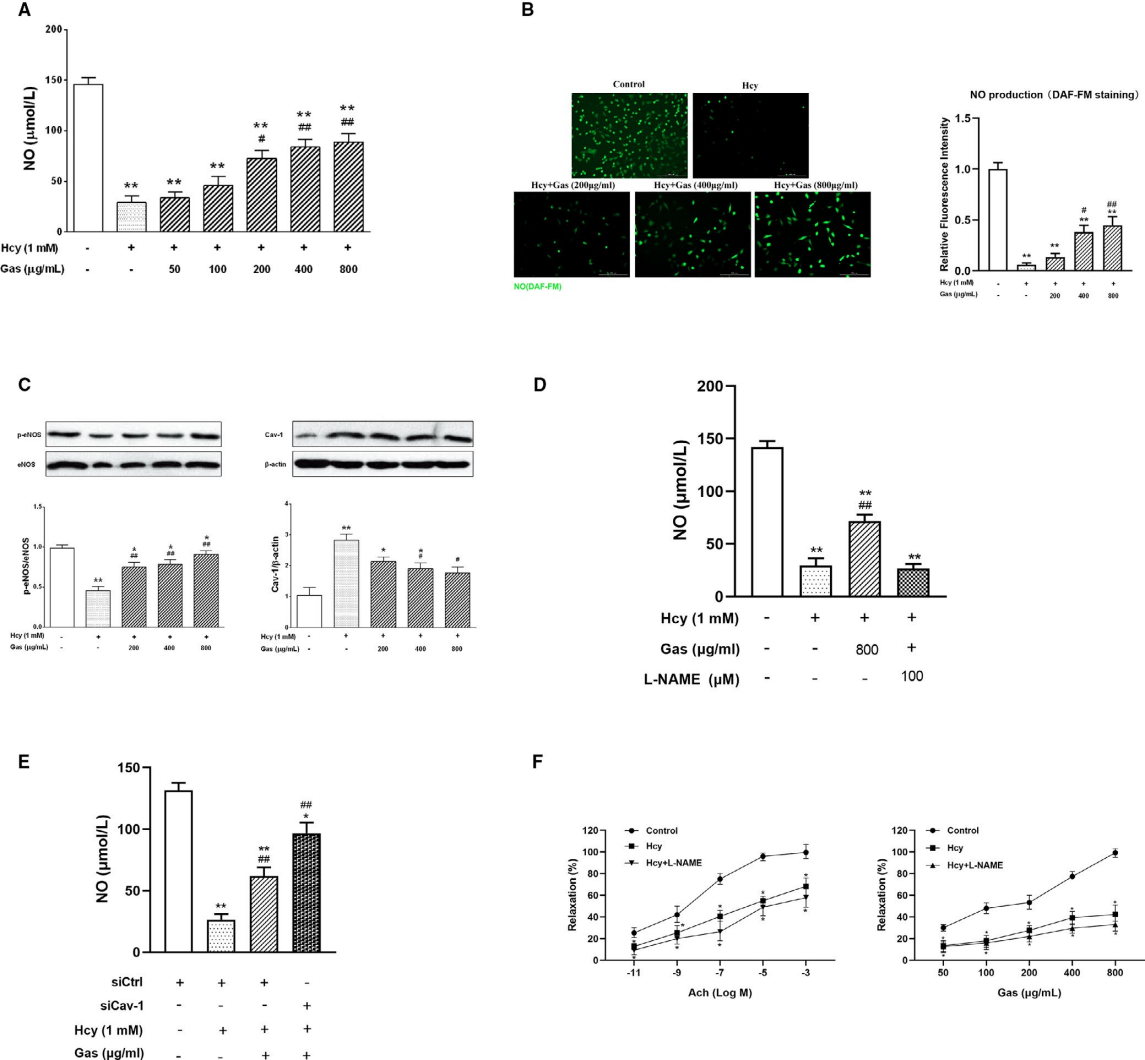
Gas decrease Hcy‐induced in NO production and increase vasodilatory effect in thoracic aorta rings dependent on Cav‐1/eNOS pathway. (A) HUVECs cultured with Hcy (1 mmol/L) were treated with Gas (50‐800 μg/mL) for 24 h, then the NO production was measured by kit assay. (B) HUVECs cultured with Hcy (1 mmol/L) were treated with Gas (200‐800 μg/mL) for 24 h, then incubated with DAF‐FM (5 μmol/L)for 30 min at 37℃. Imaged at 10 × magnification. Scale bars = 200 μm. NO levels were determined from the mean fluorescence and quantified using Image J software. (C) Quantification of Cav‐1, p‐eNOS and eNOS protein are measured by using Western blotting in Gas‐treated HUVECs with Hcy. (D) HUVECs exposed to L‐NAME (100 μmol/L) 30 min before Hcy (1 mmol/L) and Gas (800 μg/mL) treated, then the NO production was measured. (E) HUVECs were transfected with Cav‐1 siRNA or control siRNA. After 24 h, HUVECs were incubated with Gas or Hcy for another 24 h, then the NO production was measured. (F) Rings were pre‐treated with L‐NAME (100 μmol/L) or vehicle for 20 min, and then treated with Hcy, finally by Gas or Ach. Data are Mean ± SEM (three independent experiments). *P < 0.05 vs. control group. #*P* < 0.05 vs. Hcy group

To better understand the possible contribution of NO to the protective effects of Gas, we performed an NO analysis with a kit assay and fluorescence probe after the HUVECs were treated with Hcy and Gas. Compared with the control, Hcy significantly decreased NO production in the HUVECs. However, Gas (50‐800 µg/mL) treatment effectively promoted NO production, with a maximal increase at 800 µg/mL, showing a significant difference in the 200 to 800 μg/mL groups (Figure 3A, B). NO is mainly produced by eNOS in endothelial cells. Hence, we explored the involvement of the Cav‐1/eNOS pathway in the protective effects of Gas and examined the protein levels by Western blots. The results showed that Hcy addition inhibited total eNOS expression and eNOS phosphorylation at Ser‐1177, and stimulation by Gas caused a remarkable increase in eNOS phosphorylation and a significant decrease in Cav‐1 protein expression (Figure 3C). For eNOS phosphorylation, Gas significantly restored its phosphorylation level in the 200 to 800 μg/mL groups, but for the expression of Cav‐1, only the 400 and 800 μg/mL groups showed significant differences compared with the Hcy group. These results indicate the effects of Gas on NO production were associated with the Cav‐1/eNOS pathway.

To further investigate the role of the Cav‐1/eNOS pathway in the effects of Gas on NO production, we inhibited eNOS and Cav‐1 through specific inhibitors and small interfering RNAs. As shown in Figure 3D, the eNOS inhibitor L‐NAME decreased Gas‐restored NO production, and the Gas‐treated group showed a significant difference compared with the Hcy group, but after L‐NAME treatment, it showed no significant difference compared with the Hcy group. In the Cav‐1 inhibition experiment, we used Cav‐1‐specific siRNA to knock down Cav‐1 expression. The knockdown efficiency was verified by Western blots (Figure S2). NO production was detected following the knockdown of Cav‐1 with siRNA, and the results showed that Cav‐1 knockdown further elevated the effect of Gas on NO, which showed a smaller significant difference compared with that of the control group (Figure 3E).

### 3.6 Gas improved endothelium‐induced vasodilation

The ACh‐induced endothelial‐dependent vasodilatory reaction has now become a classic index of vascular endothelial cell function.^25^, ^26^ To evaluate the effect of Gas on vascular function, we assessed relaxation of thoracic aortic rings from rats and utilized L‐NAME as a specific inhibitor to further determine the involvement of eNOS in the protective effects of Gas. As shown in Figure 3F, ACh exhibited a dose‐dependent increase in endothelium‐mediated vasodilation, which was inhibited by Hcy; this effect could be suppressed by L‐NAME (left panel). Similar effects were observed with Gas, which showed a dose‐dependent effect on the aortic rings (right panel). The above data demonstrated the key role of the Cav‐1/eNOS pathway in Gas function.

### 3.7 Gas partially protected HUVECs in a PI3K/Akt pathway‐dependent manner

**FIGURE 4:**
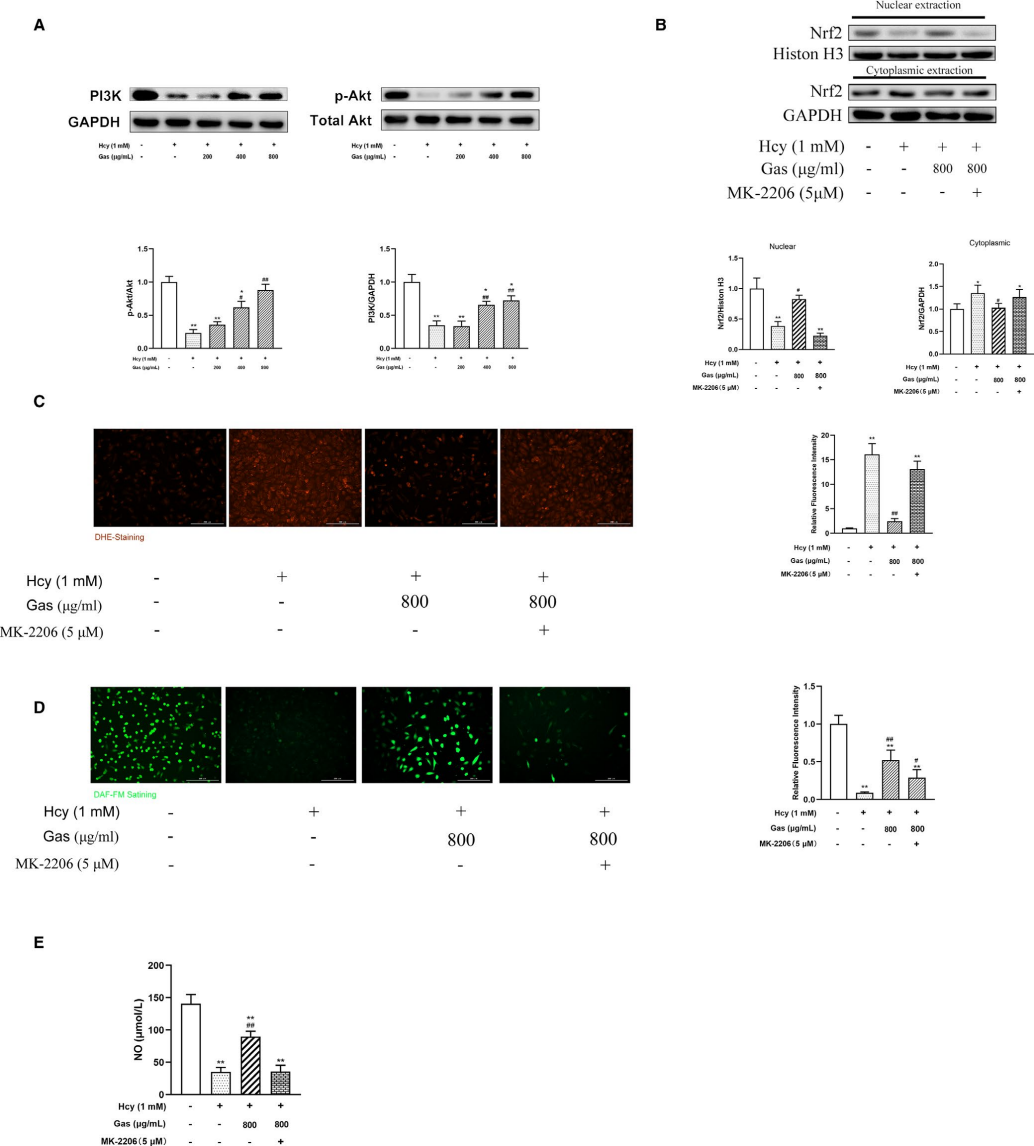
Gas partially protected the HUVECs through the PI3K/Akt pathway. (A) HUVECs cultured with Hcy (1 mmol/L) were treated with Gas (200‐800 μg/mL) for 24 h, quantification of PI3K and p‐Akt/Akt protein expression in Gas‐treated HUVECs with Hcy. (B) HUVECs exposed to MK‐2206 (5 μmol/L) with Hcy (1 mmol/L) and Gas (800 μg/mL) treated, then Nrf2 subcellular localization was determined by Western Blot. HUVECs exposed to MK‐2206 (5 μmol/L) with Hcy (1 mmol/L) and Gas (800 μg/mL) treated, then (C) ROS were detected by DHE (2 μmol/L) staining, and (D‐E) NO production was detected by DAF‐FM(5 μmol/L) staining and kit assay, respectively. Imaged at 10 × magnification. Scale bars = 200 μm. Mean fluorescence was quantified using Image J software. Data are Mean ± SEM (three independent experiments). *P < 0.05, **P < 0.01 vs. control group. #*P* < 0.05, ##*P* < 0.01 vs. Hcy group

Nrf2 and eNOS are both modulated by the PI3K/Akt signalling pathway, the activation of which plays a pivotal role in cellular homeostasis under oxidative stress.^27^ We have been suggested that Gas exerted its protective effects through a mechanism dependent on PI3K/Akt, which, based on our results above, indicated that Gas exerted its effect through the Cav‐1/eNOS and Nrf2/ARE pathways. Hence, we assessed PI3K, p‐Akt and Akt protein expression under the stimulation of Hcy with or without Gas. The results showed that the Hcy‐treated HUVECs had decreased PI3K and p‐Akt expression but did not show a change in total Akt expression, and HUVECs treated with Gas showed restored PI3K and p‐Akt expression, with significant differences in the 400 and 800 μg/mL groups (Figure 4A).

To explore the role of the PI3K/Akt pathway in the protective effects of Gas, we incubated the Akt inhibitor MK‐2206 (5 μmol/L) with the cells in treatment of Gas (800 μg/mL) and Hcy (1 mmol/L). First, we treated HUVECs with MK‐2206 in the presence or absence of Hcy and Gas. Next, we tested the Nrf2 distribution in the cytoplasm and nucleus and the production of ROS in HUVECs to investigate whether Gas ameliorated oxidative stress in a mechanism dependent on Akt‐mediated Nrf2 nuclear localization. The results showed that Hcy decreased the expression of Nrf2 in the nucleus and that treatment with Gas improved its expression, which was blocked by an Akt inhibitor (Figure 4B). Moreover, the protective effect of Gas mediated through inhibition of Hcy‐induced ROS production was similarly abolished by the Akt inhibitor (Figure 4C), and this group showed no significant difference compared with the Hcy group.

In addition, we investigated whether the PI3K/Akt pathway was involved in the promotion of NO by Gas. The results showed that the effects of Gas on NO production were inhibited by MK‐2206 (Figure 4D). Consistently, the Akt inhibitor group showed a lower number of green foci, indicating lower NO production detected by the fluorescence probe than that of the Gas‐treated group (Figure 4E). The results above indicated that Gas partially protected HUVECs in a mechanism dependent on the PI3K/Akt pathway.

### 3.8 Gas treatment alone partially activated the PI3K/Akt/eNOS and Nrf2/ARE pathways

To investigate the effects of Gas alone on the levels of PI3K/Akt/eNOS and the Nrf2/ARE pathway, we treated HUVECs with Gas alone, and the related protein expression was examined. The results showed that the effects of Gas alone on PI3K/Akt resulted in no significant difference in PI3K expression, while p‐Akt only in 800 μg/mL resulted in a significant difference (Figure S3A). In addition, Gas treatment alone did not change the expression of total eNOS and Cav‐1 expression but enhanced p‐eNOS at 400 and 800 μg/mL, which demonstrated that Gas could phosphorylate eNOS and restore the production of NO (Figure S3B). Regarding the changes in cytosolic and nuclear Nrf2 protein levels, only Gas at 400 and 800 μg/mL increased Nrf2 nuclear translocation (Figure S3C). Gas alone also elevated HO‐1, SOD‐1 and catalase expression and resulted in the greatest increase at 800 μg/mL (Figure S3D). The above results validate that the combination of Hcy and Gas may induce those proteins, most but not all of which can be induced with Gas alone. In addition, p‐Akt and Nrf2 nuclear translocation was only induced by the 800 μg/mL treatment.

## 4 DISCUSSION

**FIGURE 5:**
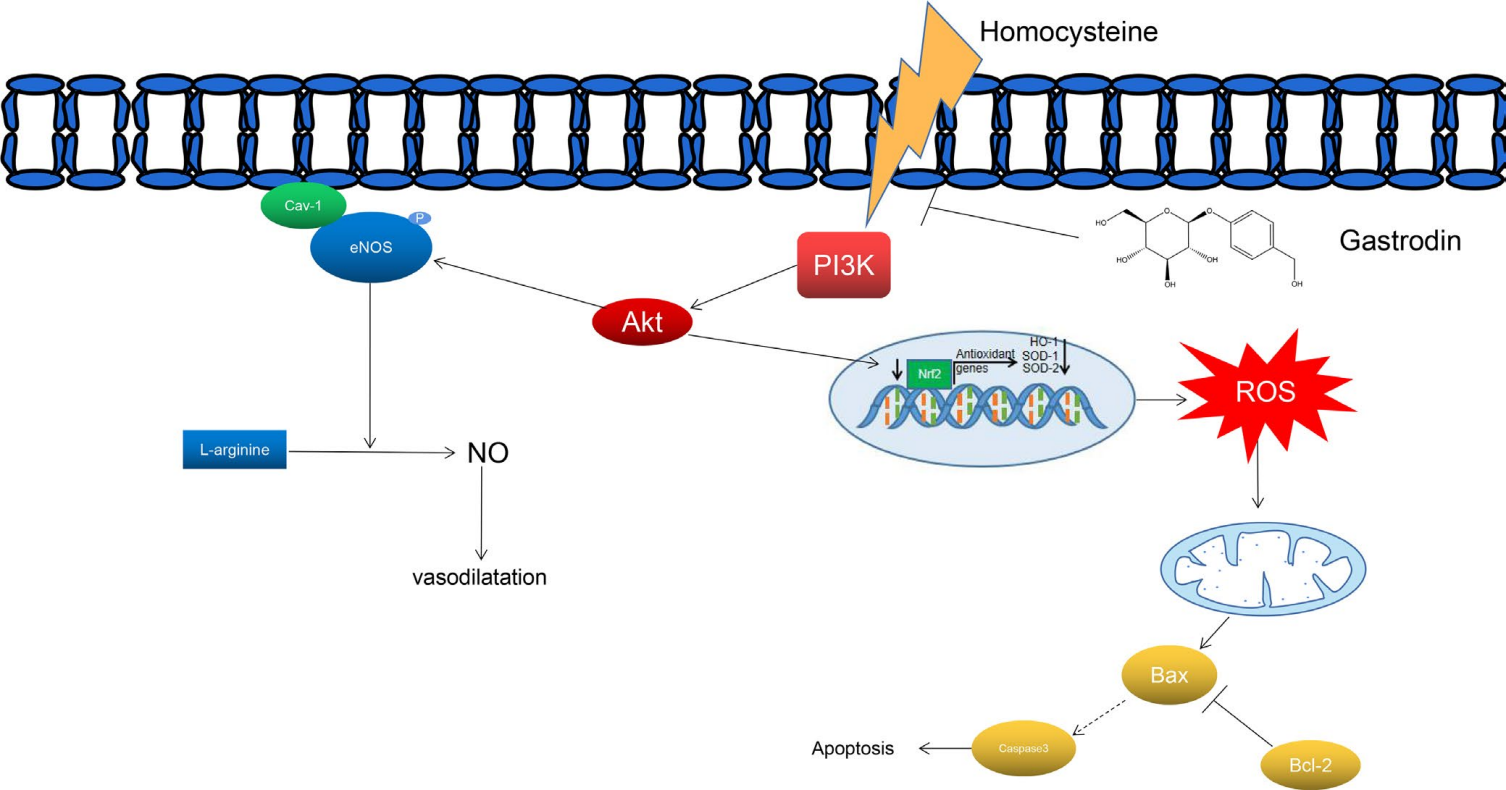
Model for Gas ameliorated endothelial cell damage partly through PI3K/Akt/eNOS and Nrf‐2/ARE pathway

In this study, we investigated the protective effects of Gas on Hcy‐mediated injury in HUVECs. The salient findings of this study are as follows: (1) Hcy treatment caused a decrease in cell viability and total antioxidant capacity and an increase in MDA, LDH and ROS levels, and Gas markedly ameliorated the Hcy‐mediated injury. (2) Gas treatment restored the Hcy‐induced Nrf2 transfer from the nucleus to the cytoplasm and decreased HO‐1, SOD‐1 and catalase, the downstream targets of Nrf2. (3) Gas recovered the Hcy‐mediated reduction in NO production of HUVECs through the PI3K/Akt/eNOS pathway. (4) Gas treatment inhibited Hcy‐induced HUVEC apoptosis. (5) Inhibition of the PI3K/Akt pathway abolished the protective effects of Gas. (6) Hcy addition significantly decreased the relaxation of thoracic aortic rings, which was improved by Gas through the regulation of Cav‐1/eNOS. These findings indicated that Gas ameliorated endothelial cell damage partly through the PI3K/Akt/eNOS and Nrf2/ARE pathways (Figure 5).

Several studies have reported that Hcy regulates the pathophysiological process of cardiovascular diseases such as atherosclerosis and hypertension.^28^, ^29^ The Hcy‐induced model of endothelial cell damage was reported in the early 1980s.^30^ Hcy induced endothelial dysfunction, leading to oxidative inactivation of NO and ROS generation,^31^, ^32^ thus promoting cardiovascular disease. Montezano et al reported that Hcy could cause endothelial damage in which various markers of damage, such as MDA and LDH, could play a notable role.^33^ Consistent with these findings, we found that after Gas treatment, the Hcy‐induced effects on cell viability and T‐AOC were restored, and the Gas‐treated HUVECs exhibited a significant decrease in the MDA, LDH and ROS levels. Taken together, these results indicated that Gas could protect the Hcy‐treated HUVECs by restoring T‐AOC and regulating MDA, LDH and ROS production.

In addition, many studies have proven that excessive ROS cause apoptosis, and we found that Gas can dose‐dependently inhibit the Hcy‐induced HUVEC apoptosis. These findings suggested that the potential protective mechanisms of Gas on Hcy‐induced HUVEC damage might be responsible for the decrease in markers of damage and the reduction in apoptosis. Overexpression of bax and reduction of bcl‐2 result in activation of downstream caspase 3, ultimately triggering cell apoptosis.^34^ Ataie et al found that Hcy‐induced overexpression of bax and cleaved caspase 3.^35^ Our findings revealed that Gas inhibited the expression of bax and cleaved caspase 3 while increasing the expression of bcl‐2. Hence, we speculated that Gas attenuated the Hcy‐induced HUVEC apoptosis through the regulation of the ratio of bax/bcl‐2 and the reduction of cleaved caspase 3.

Moreover, high levels of Hcy, increased oxidative stress and reduced antioxidant function have been related to the risk of cardiovascular disease.^36^ Previous work on Hcy has indicated that the downstream enzymes of Nrf2 are significantly decreased and ROS are increased after Hcy exposure.^37^ Therefore, we investigated whether Gas could counteract the increase in oxidative stress caused by Hcy by regulating the Nrf2/ARE pathway. Our data were similar to those of Elanchezhian's study, which showed that Hcy reduced the downstream enzymes of Nrf2 at the protein and mRNA levels;^37^ furthermore, we found that Gas could restore the expression of these enzymes, which might be responsible for the regulation of Nrf2 nuclear translocation.

Currently, NO is believed to play an important regulatory role in normal physiological processes, and Hcy can enhance ROS production, which is responsible for the decrease in NO levels, leading to endothelial dysfunction.^38^ Our data demonstrated that Gas promoted the generation of NO and inhibited the production of ROS, indicating that Gas alleviated Hcy‐induced oxidative stress in HUVECs. To explore the cause of the NO reduction, we measured the expression of Cav‐1 and eNOS, which are two crucial enzymes in NO production. Cav‐1, the major isoform of caveolin, negatively regulates eNOS, leading to inhibition of NO production and accumulation of ROS, thus causing cellular damage.^39^ In the present study, we found that treatment with Hcy alone in HUVECs resulted in increased Cav‐1 expression. Additionally, exposure of HUVECs to Gas could restrain Cav‐1 protein expression. However, Meye's study showed that Hcy decreases Cav1 levels, which contrasts with our results.^7^ We believe that a major reason for this discrepancy is the different cell models used, which suggests that the mechanism of Hcy‐induced endothelial injury is different in different cells. Consistently, another study on Hcy‐induced endothelial injury reached a similar conclusion to that of our study: Hcy induced an increase in the expression of Cav‐1 in HUVECs.^40^

Studies in eNOS‐deficient mice found that a lack of the eNOS gene led to systemic vascular endothelial dysfunction, significantly decreased vasodilator effects and even severe hypertension.^41^ In our study, Gas increased the eNOS protein levels, as well as p‐eNOS at Ser‐1177, which was consistent with other studies showing that eNOS had protective effects against Hcy‐induced endothelial dysfunction.^42^ Therefore, we established that the reduction of Cav‐1 and promotion of eNOS phosphorylation may contribute to the ability of Gas to attenuate HUVEC injury. However, the eNOS inhibitor L‐NAME restricted the NO increase induced by Gas. These results suggested that Hcy caused endothelial cell damage, which was ameliorated by Gas via Cav‐1/eNOS regulation.

Many cell processes are regulated by the PI3K/Akt signalling pathway.^43^ Akt, also known as protein kinase B, is a key kinase in the PI3K/Akt signalling pathway. Activated Akt regulates cell function by phosphorylating downstream factors such as enzymes, kinases and transcription factors.^44^ The PI3K/Akt/eNOS signalling pathway regulates NO production through the phosphorylation of eNOS at Ser‐1177 under various stimuli.^45^ In addition, phosphorylation of Akt promoted Nrf2 nuclear translation and activated downstream enzymes.^46^ Therefore, we blocked the phosphorylation of Akt by MK‐2206 and found that the protective effects of Gas were abolished by the reduction of ROS, promotion of NO and nuclear translation of Nrf2.

Our study confirmed that PI3K/Akt/eNOS and Nrf2/ARE may be crucial in the protective effects on endothelial function mediated by Gas. The biological relevance of these findings was shown by the ability of Gas to induce vasodilation in thoracic aortas. Thus, we can presume that Gas could have a positive impact on clinical conditions of cardiovascular diseases in which Hcy is up‐regulated, such as hyperhomocysteinemia, arterial stiffness, diabetes and hypertension.^47^

One of the limitations of our study is that we used the HUVEC line to evaluate the protective effects of Gas. Primary cells isolated from the human umbilical vein or human arterial are more desirable experimental materials than cell lines for investigating pharmacologic effects. Second, we did not examine the protective mechanisms of Gas in animal models. However, Liu et al found that the vascular endothelial dysfunction induced by hyperhomocysteinemia in rats can be prevented through a decrease in MDA levels, increase in NO production and mitigation of Hcy‐induced damage as well as other protective effects.^48^ In addition, activation of the eNOS signalling pathway was shown to inhibit Hcy‐induced endothelial dysfunction in an animal model.^49^ Furthermore, in type 1 diabetic rats, overactivation of Cav‐1 contributes to aggravated endothelial dysfunction and hypertension.^50^ These findings suggest a protective mechanism against Hcy‐induced damage in vivo, thereby validating our results.

## 5 CONCLUSION

In summary, our study demonstrated that Gas could have a positive impact on the cell viability of HUVECs treated with Hcy, resulting in a significant decrease in the MDA, LDH, and ROS levels and an increase in the NO content and T‐AOC. Gas could induce vasodilation in thoracic aortas. Moreover, Gas attenuated Hcy‐induced HUVEC apoptosis through regulation of bax, bcl‐2 and cleaved caspase 3. These effects correlated with the increased phosphorylation of Akt and augmented eNOS and Nrf2 activation. Altogether, our findings suggested that Gas, a natural compound, could be a potential lead compound for the treatment or remission of some cardiovascular diseases, which appears to depend mainly on the PI3K/Akt/eNOS and Nrf2/ARE pathways.

## CONFLICT OF INTEREST

The authors declare there is no conflicts of interest regarding the publication of this paper.

## AUTHOR CONTRIBUTION

**Jiyu Chen:** Data curation (equal); Methodology (lead); Project administration (lead); Validation (lead). **Yanli Huang:** Formal analysis (lead); Methodology (supporting); Validation (supporting); Writing‐original draft (lead). **Xiaochuan Hu:** Data curation (supporting); Software (equal); Writing‐original draft (supporting). **Xiaohong Bian:** Funding acquisition (equal); Writing‐review & editing (equal). **sihui nian:** Conceptualization (lead); Funding acquisition (equal); Writing‐review & editing (equal).

## Supporting information

Fig S1

Fig S2

Fig S3

Figs S1‐S3
