# Supplementary figures and images for: Gastrodin prevents homocysteine‐induced human umbilical vein endothelial cells injury via PI3K/Akt/eNOS and Nrf2/ARE pathway
Source: J Cell Mol Med. 2020 Dec 15;25(1):345–57. doi: 10.1111/jcmm.16073 (PMC7810955; doi:10.1111/jcmm.16073)

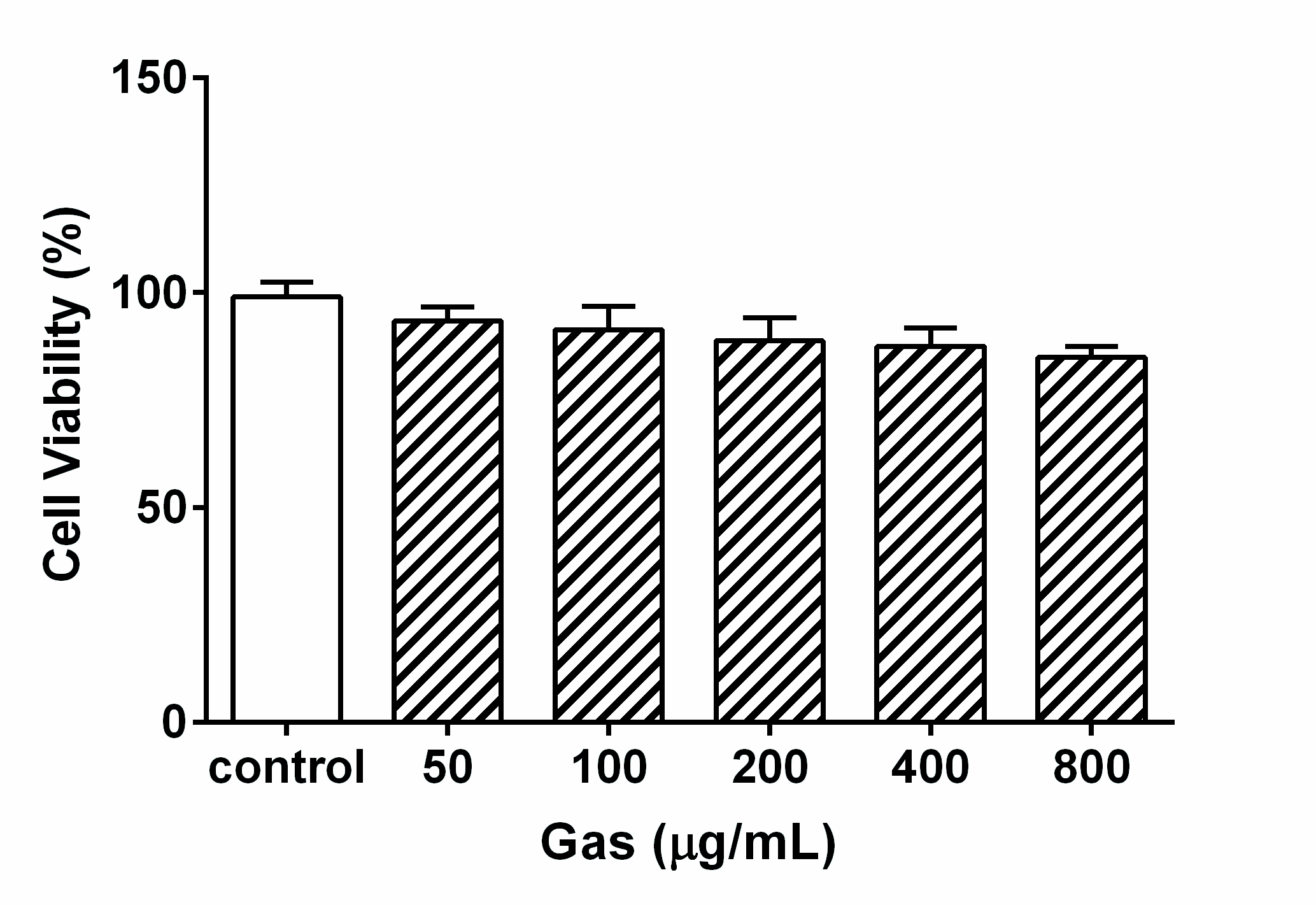

Supplement: Supplementary file 1 — Fig S1 [file JCMM-25-345-s001.tif]

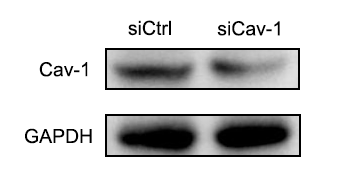

Supplement: Supplementary file 2 — Fig S2 [file JCMM-25-345-s002.tif]

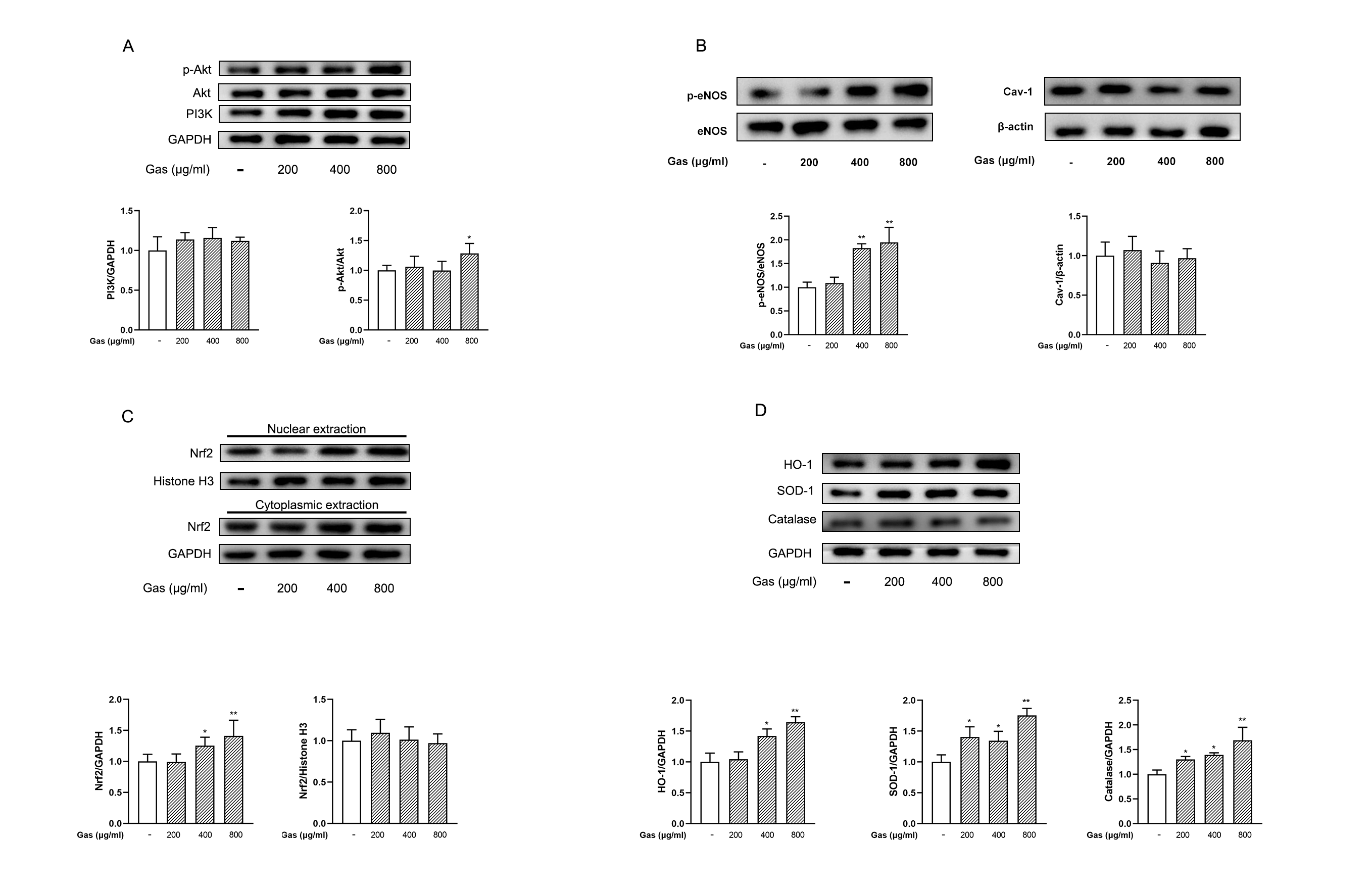

Supplement: Supplementary file 3 — Fig S3 [file JCMM-25-345-s003.tif]
