# Supplementary material for: Gastrodin prevents homocysteine‐induced human umbilical vein endothelial cells injury via PI3K/Akt/eNOS and Nrf2/ARE pathway
Source: J Cell Mol Med. 2020 Dec 15;25(1):345–57. doi: 10.1111/jcmm.16073 (PMC7810955; doi:10.1111/jcmm.16073)
Supplement: Supplementary file 4 — Figs S1‐S3 [file JCMM-25-345-s004.doc]

**Figure S1.** Gas performed less cytotoxicity. HUVECs were incubated for 24 h in the presence of Gas (50-800 µg/mL). Cell viability was measured by MTT assay. A representative viability is shown above the graph. Data are Mean ± S.E.M (three independent experiments). There is no statistical differentiation between Gas-treated groups and control group.

**Figure S2.** Knockdown efficiency of Cav-1 siRNA. HUVECs were transfected with Cav-1 siRNA or control siRNA, and the silencing efficiency of Cav-1 siRNA was determined by Western blotting.

**Figure S3.** Incubation of Gas alone partly activated PI3K/Akt/eNOS and Nrf2/ARE pathway. HUVECs were incubation with Gas alone for 24 h, PI3K/Akt/eNOS and Nrf2/ARE pathway were determined by Western blotting. (A) Quantification of PI3K and p-Akt/Akt protein expression. (B) Quantification of Cav-1 and eNOS protein expression (C) Quantification of Nrf2 subcellular localization. (D) Quantification of Nrf2/ARE pathway downsrtream enzymes SOD-1, HO-1 and Catalase protein expression. Data are Mean ± S.E.M (three independent experiments). *P < 0.05, **P < 0.01 vs. control group.
